# Imaging Reactive Oxygen Species-Induced Modifications in Living Systems

**DOI:** 10.1089/ars.2015.6415

**Published:** 2016-06-01

**Authors:** Giuseppe Maulucci, Goran Bačić, Lori Bridal, Harald H.H.W. Schmidt, Bertrand Tavitian, Thomas Viel, Hideo Utsumi, A. Süha Yalçın, Marco De Spirito

**Affiliations:** ^1^ Institute of Physics, Catholic University of Sacred Heart, Roma, Italy.; ^2^ Faculty of Physical Chemistry, University of Belgrade, Belgrade, Serbia.; ^3^ Laboratoire d'Imagerie Biomédicale, Sorbonne Universités and UPMC Univ Paris 06 and CNRS and INSERM, Paris, France.; ^4^ Department of Pharmacology and Personalised Medicine, CARIM, Faculty of Health, Medicine & Life Science, Maastricht University, Maastricht, the Netherlands.; ^5^ Laboratoire de Recherche en Imagerie, Université Paris Descartes, Hôpital Européen Georges Pompidou, Service de Radiologie, Paris, France.; ^6^ Innovation Center for Medical Redox Navigation, Kyushu University, Fukuoka, Japan.; ^7^ Department of Biochemistry, School of Medicine, Marmara University, İstanbul, Turkey.

## Abstract

***Significance:*** Reactive Oxygen Species (ROS) may regulate signaling, ion channels, transcription factors, and biosynthetic processes. ROS-related diseases can be due to either a shortage or an excess of ROS. ***Recent Advances:*** Since the biological activity of ROS depends on not only concentration but also spatiotemporal distribution, real-time imaging of ROS, possibly *in vivo*, has become a need for scientists, with potential for clinical translation. New imaging techniques as well as new contrast agents in clinically established modalities were developed in the previous decade. ***Critical Issues:*** An ideal imaging technique should determine ROS changes with high spatio-temporal resolution, detect physiologically relevant variations in ROS concentration, and provide specificity toward different redox couples. Furthermore, for *in vivo* applications, bioavailability of sensors, tissue penetration, and a high signal-to-noise ratio are additional requirements to be satisfied. ***Future Directions:*** None of the presented techniques fulfill all requirements for clinical translation. The obvious way forward is to incorporate anatomical and functional imaging into a common hybrid-imaging platform. *Antioxid. Redox Signal*. 24, 939–958.

## Introduction

Reactive oxygen species (ROS) such as superoxide, hydrogen peroxide, and peroxynitrite are highly reactive in terms of oxidative modifications of biomacromolecules. Exogenous ROS can be produced from pollutants, tobacco, smoke, drugs, xenobiotics, or radiation; whereas endogenous ROS are produced intracellularly through multiple mechanisms. Depending on the cell and tissue types, the major sources are NADPH oxidase (NOX) complexes (seven distinct isoforms) in cell membranes, mitochondria, peroxisomes, and endoplasmic reticulum (30).

**FIG. 1.**
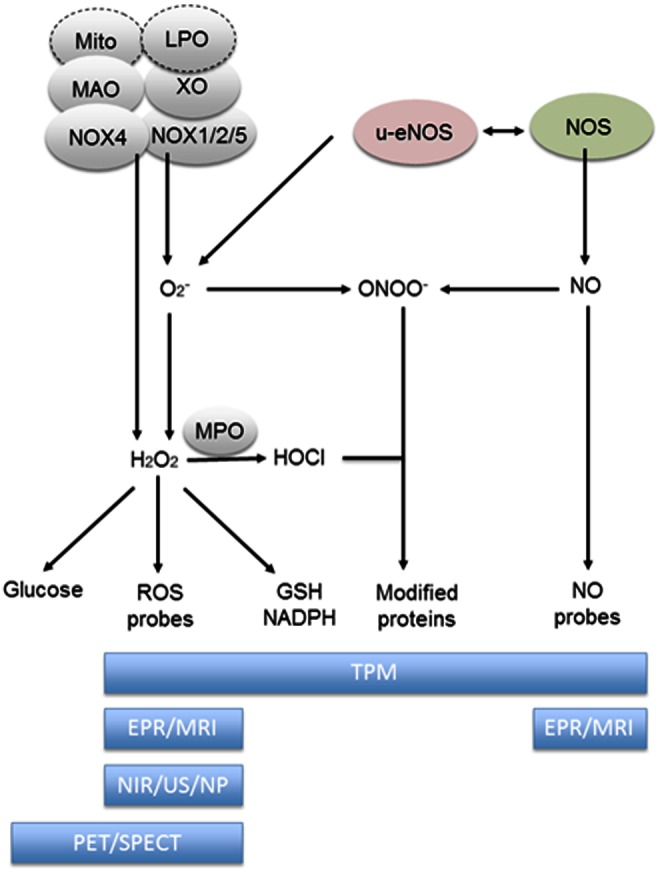
**Spectrum of different ROS imaging techniques.** In the *upper* part, different sources of ROS are shown: Mitochondria (mito), lipid peroxides (LPO), monoamine oxidase (MAO), nicotinamide adenine dinucleotide phosphate oxidase (NOX4 and NOX 1/2/5), xanthine oxidase (XO), and nitric oxide synthases (NOS and e-NOS). These result in different types of ROS [including superoxide radical (O_2_^•−^:), hydrogen peroxide (H_2_O_2_), hypoclorous acid (HOCl), peroxynitrite radical (ONOO^−^:), nitric oxide(NO)] and ROS-induced modifications of GSH, NADPH, proteins, or glucose uptake, which, in turn, are detected by different imaging technologies (for abbreviations and details, see text). ROS, reactive oxygen species. To see this illustration in color, the reader is referred to the web version of this article at www.liebertpub.com/ars

Mitochondria produce superoxide radical (O_2_^•−^) when oxygen is prematurely and incompletely reduced. Superoxide can initiate lipid peroxidation in its protonated form, hydroperoxyl HO_2_·, and can be converted to hydrogen peroxide (H_2_O_2_). Myeloperoxidase (MPO), which is released from cytoplasmic granules of activated phagocytes by a degranulation process, reacts with H_2_O_2_ and chloride ions to generate hypochlorous acid/hypochlorite (HOCl/OCl(-)). HOCl, a strong oxidant, in turn, reacts with proteins to form HOCl-modified proteins. Reactive nitrogen species (RNS) are derived from nitric oxide (NO^•^) and superoxide (O_2_^•−^) *via* the enzymatic activity of inducible nitric oxide synthase (NOS) and NOX, respectively. The reaction of nitric oxide (NO^•^) with superoxide (O_2_^•−^) leads to the formation of peroxynitrite (ONOO^−^) (Fig. 1) (30).

These reactive species are essential regulators of several physiological processes, ranging from intermediary metabolism to the inflammatory response. Their altered spatiotemporal distribution plays a central role in the physiopathology of disease (21).

An understanding of the complexity of ROS signaling requires the determination of their spatiotemporal distribution with high resolution, specificity, and sensitivity. Toward this aim, significant progress in ROS imaging at the level of intact cells, tissues and whole organs, as well as living organisms was achieved in the previous decade. Among these advancements, an important role was played by the development of novel synthetic or genetically encoded fluorescent ROS indicators and *in vivo* imaging technologies (17, 91, 96, 123, 127, 145).

In particular, the possibility of detecting ROS dynamics *in vivo* has stimulated research in medical imaging with the aim of providing new information that will be beneficial for disease management. This area of medical imaging research covers a wide domain of different imaging modalities, each with its own sensitivity and resolution. Modalities include Magnetic Resonance Imaging (MRI), Ultrasound (US), Positron Emission Tomography (PET), Single-Photon Emission Computed Tomography (SPECT), and other optical imaging methods (2, 51, 103, 108, 109, 138) (Fig. 1). In this context, improvements in detection efficiency as well as new contrast agents for these well-established modalities were developed in the previous decade. However, low levels of intracellular ROS require new and more sensitive methods. Here, we will review the methods emerging to image the complexity of the ROS dynamics *in vivo* with a focus on those that have potential for clinical application.

## Redox-Sensitive Two-Photon Microscopy

Two-photon microscopy (TPM) is a well-established sub-micron resolution imaging technique that is characterized by low phototoxicity and deep tissue penetration. In the two-photon process, the probe absorbs two photons whose individual energy is only half of the energy needed to excite that molecule (109). TPM excitation *via* near infrared (NIR) laser light reduces tissue and water absorption. Penetration depths can reach 1 mm into biological tissues, and the reduction of photo-bleaching, photo-damage, and phototoxicity is achieved by the spatial confinement of excitation (109). With these advantages, TPM has yielded novel and unique structural and functional information on cells and tissues.

In this context, determination of the spatial distribution of redox-active compounds and their time evolution is an important issue to be addressed, since redox homeostasis, playing a crucial role in many pathologies, can be a decisive target for pharmaceutical intervention (30).

Several fluorescence approaches to image ROS and redox potentials with high resolution have been attempted to address this task. Two of them are the most promising: Two-photon Fluorescence Ratio Imaging Microscopy (TP-FRIM) and Two Photon Fluorescence Lifetime Imaging Microscopy (TP-FLIM) (94, 137, 143). These techniques have allowed a quantification of specific concentrations of intracellular redox species by canceling out the possible perturbations due to instrument efficiency and dye concentration.

In TP-FRIM, the absorption or emission spectrum is differently sensitive to the redox state of the compound. One wavelength range of the emission or excitation spectrum may be less sensitive, or sensitive in the opposite direction with respect to another selected range. Because absorption or emission originates from the same volume, the ratio of fluorescence measured in the two ranges is independent of optical path length, probe concentration, and excitation intensity.

TP-FLIM allows for the detection of the redox state of compounds by measuring differences in the exponential decay rate of the fluorescence (lifetime) of the probe by single-wavelength excitation. A quantitative determination of the redox state independent of probe concentration could be obtained.

### Two-photon redox-sensitive probes

Determination of the spatial distribution of different redox-active compounds (GSH, NAD(P)H, H_2_O_2_, NO *etc*.) is an important aim for diagnosis and treatment. Although a number of probes that are able to detect fluorescence in cultured cells are available, recent efforts have aimed at developing specific and highly sensitive TP-FRIM and TP-FLIM-based probes to improve a quantitative analysis of ROS in deep tissue and for intra-vital microscopy (17, 19, 27, 79, 126, 145).

### Two-photon sensitive probes for assessment of glutathione redox state

The redox state of the reduced and oxidized glutathione couple (GSH:GSSG), the most abundant redox couple in a cell, is an informative readout of the cellular redox environment (30). Glutathione-specific, redox-sensitive variants of the Yellow Fluorescent Protein (rxYFP) and the Green Fluorescent Protein (roGFP1 and roGFP2) allowed FRIM real-time monitoring in the intracellular GSH:GSSG redox ratio (92–94, 123). The specificity of these probes for glutathione was enhanced by linking them to human glutaredoxin 1 (Grx1) (12). To extend *in vivo* use of these probes, Wolf *et al.* (145) generated transgenic mice expressing roGFP in several tissues to ratiometrically monitor oxidative stress in skin epidermal keratinocytes. However, visible excitation and emission light do not permit a deeper penetration in tissues. As a result, measurement of the cellular glutathione redox potential (EG) is affected by non-negligible systematic errors (95). Furthermore, these redox probes, when linked to enzymatically active redox proteins (*i.e*., Grx1), may alter cellular redox homeostasis. Guzman and coworkers reported measurements of mitochondrial oxidative stress on dopaminergic neurons in transgenic mice expressing mito-roGFP, an roGFP that selectively tags targeted to mitochondria, with TPM (42). However, the suitability of this probe for potential TP-FRIM application has yet to be tested. To overcome these issues, several GSH-sensitive, TP-excitable, nonencoded chemoselective probes were engineered for *in vivo* applications (79). However, even in these cases, TP-FRIM/TP-FLIM potentials have yet to be characterized. Besides all these profuse efforts, further improvements in the development of glutathione-specific redox probes are still needed.

### Two-photon NADPH redox state sensitive probes

**FIG. 2.**
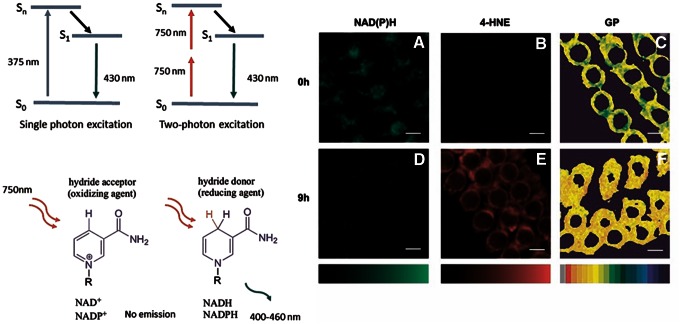
**Acoustic trauma induces NAD(P)H oxidation, lipid peroxidation, and loss of membrane fluidity.** NAD(P)H can be excited by a one-photon process: For example, it can absorb one photon at 375 nm, and emit one photon at 430 nm. In the two-photon process, NAD(P)H absorbs two photons of 750 nm whose individual energy is about one half of the energy needed to excite that molecule. NAD(P)H does not emit fluorescence in its oxidized state. **(A)** Representative fluorescence NAD(P)H images at different time points (*n* = 5 animals per time point) after the trauma. **(B)** 4-HNE assays at different times after acoustic trauma. **(C)** Fluidity maps at different times after acoustic trauma. **(D)** Reduced NAD(P)H percentages at different times after the trauma. From the figure, the topologically differentiated NAD(P)H oxidation is also evident on the *outer*, *middle*, and *inner rows* of OHCs. **(E)** 4-HNE concentrations at different times after acoustic trauma. **(F)** GP values of hair bundle region (maximum of the GP profiles) at different times after the trauma. Adapted from Maulucci *et al*. (96). Reprinted with permission of Elsevier. NADPH, nicotinamide adenine dinucleotide phosphate. To see this illustration in color, the reader is referred to the web version of this article at www.liebertpub.com/ars

The intracellular metabolic substrates NADH and NADPH (NAD(P)H) have been used as intrinsically fluorescent probes for metabolic states, cancer detection, and tissue oxygen supply, allowing label-free *in vivo* imaging of tissues (17, 90a, 124, 125, 130). NAD(P)/NAD(P)H auto-fluorescence can, therefore, be detected and related to other different physical quantities to gain further details on insight into the processes regulating redox homeostasis. For example, the mechanism by which noise-induced ROS mediates an impairment in acoustic recovery capacity was elucidated by relating intracellular distribution of NAD(P)H in a noise-stressed mammalian cochlea to the generation of lipid peroxides and to the spatial organization of lipids inside membranes. This allowed a disclosure of the mechanism of noise-induced ROS production and impairment in the acoustic recovery capacity (Fig. 2) (96). Skala *et al.* (127) combined cellular redox ratio, NAD(P)H and FAD lifetime, and subcellular morphology to quantitatively detect NAD(P)H. With this approach, metabolic and structural modifications at the earliest stages of cancer development have been identified in several epithelial tissues *in vivo*. Moreover, cell Phasor, a label-free, fit-free, and sensitive innovative method that allows classification of metabolic states of cells during differentiation, has been developed from TP-FLIM data (129). Zhuo *et al.* (17) used two-photon autofluorescence and second harmonic generation (SHG) microscopy to monitor cancer progression and to classify normal and dysplastic human colonic tissues. Overall, these findings demonstrate that auto-fluorescence can provide structural and functional information for the diagnosis and therapy of pathologic epithelial tissues.

### Two-photon H_2_O_2_-sensitive probes

Hydrogen peroxide plays a key role as a cellular second messenger in a variety of signal transduction processes (30). Genetically encoded fluorescent proteins HyPer, HyPer-3, and roGFP2-Orp1 enabled transient live-cell imaging and allowed high-resolution H_2_O_2_ imaging with high specificity (41). However, these probes are applicable only to single-photon FRIM or FLIM and have limited application *in vivo*, since they may alter redox homeostasis and are genetically encoded. Several TP probes have been generated to monitor the production of intracellular H_2_O_2_ (41), but their potential TP-FRIM applications have yet to be tested. For the TP-FRIM imaging approach, a promising probe is Peroxy Naphthalene 1 (PN1). This probe can be excited at 750 nm, and it has high photostability and negligible toxicity. It also allows the determination of H_2_O_2_ distribution in live cells and tissue by TPM (19).

### Two-photon NO-sensitive probes

Many of the NO-sensitive probes are not reversible sensors, as they form covalent bonds with NO^•^. Genetically encoded FRET-based proteins allow high-resolution NO imaging in cell-based experiments (122). However, *in vivo* applications of these probes are very limited. For NO detection in an *in vivo* context, a TPM probe (QNO) with high selectivity, low cytotoxicity, pH insensitivity, and long-wavelength emission has been designed (27). QNO is composed of a quinoline derivative as the fluorophore and an *o*-phenylenediamine moiety as the receptor for NO, linked with glycinamide. The probe responded to NO over a linear range from 0.4 to 3.4 μ*M* with a detection limit of 0.084 μ*M*. QNO detects NO in living cells and tissues at a depth of 180 μm. However, TP-FRIM/FLIM properties are still not tested and further improvements in the development of NO-specific redox probes are needed.

## Chemiluminescent Imaging of ROS *In Vivo*

### NIR fluorescence and chemiluminescence

Over the previous decade, substantial progress has been made in the noninvasive real-time assessment of reactive oxygen and nitrogen species in biological systems. Bioimaging methods based on fluorescence and reaction-based approaches have received most attention, due to their ease of use, sensitivity, and selectivity to different reactive species, including reactive oxygen, nitrogen, and sulfur species. A key interest in this rapidly growing field has been the development of chemoselective probes, that is, probes that are diagnostic for a single reactive species. A great number of different reaction schemes have been exploited toward achieving this goal [reviewed in Chan *et al.* (16)], as summarized in Table 1. Moreover, the reaction-based monitoring of selective species can be combined with targeting of the probe to specific cellular organelles, as exemplified by the boronate MitoPY1 for the imaging of mitochondrial H_2_O_2_ (24).

**Table 1.** Diagnostic Probes for a Single Reactive Species

*Reactive species*	*Subclassification*	*Structure*	*Biological Half-life(s)*	*Reference*
Hydrogen peroxide	ROS	H_2_O_2_	10^−5^	(38)
Hydroxyl radical	ROS	HO•	10^−9^	(20, 38)
Hypochlorous acid	ROS	HOCl	unknown	unknown
Nitric oxide	RNS	NO	10^−3^÷1	(63, 106, 112, 70a)
Peroxyl radical, including alkylperoxyl and hydroperoxyl radicals (wherein R = H)	ROS	ROO•	10^−1^÷1	(20)
Peroxynitrite anion	RNS	ONOO^−^	0^−2^÷1	(6, 106)
Superoxide anion	ROS	•O_2_^−^	0^−6^	(38, 62)

ROS, reactive oxygen species.

Although fluorophores have been used widely for cellular imaging of reactive species, they have a number of limitations that restricts their successful application to tissues and animals. For the latter, fluorophores with absorption and emission maxima in the near-infrared region (650–900 nm) are required to maximize tissue penetration and, at the same time, to minimize interference from auto-fluorescence and hemoglobin absorption. Nagano and co-workers recently synthesized the near-infrared fluorescent probe FOSCY-1 to monitor reactive species in a mouse model of peritonitis (105). As this probe reacts with several biologically relevant reactive species, it provides general information about the presence of oxidative events rather than about the participation of specific reactive species *in vivo*. In a further development, the same group designed and synthesized novel far-red to near-infrared probes based on Si-rhodamine to selectively and noninvasively monitor HOCl in real time in mice suffering from peritonitis (70). Judged by the advances over the previous decade, it can be reasonably expected that the development of additional reactive species-specific and -nonspecific far-red to near-infrared fluorescent probes will progress rapidly.

Reaction-based methods to detect reactive species are also applicable to *in vivo* imaging modalities other than fluorescence. Chemiluminescence, for example, was used to monitor oxidative events *in vivo*. Perhaps the most commonly used probe is L-012, an analog of luminol (5-amino-2,3-dihydro-1,4-phthalazinedione) that produces much stronger signals than luminol, lucigenin, or MCLA (59). L-012 has been used successfully to noninvasively image different inflammatory processes in mice [26–28]. The probe reacts with several highly reactive species rather than being specific, for example, for O_2_^•−^, even though L-012-derived luminescence was abolished in mice lacking phagocyte NOX activity (66).

Although much emphasis is placed on chemoselective bioimaging, reactive species in biological systems likely exist as mixtures of rapidly interconverting species. As a result, the biological relevance of a single imaged species is difficult to assess, even if its presence correlates with the biological process studied, as exemplified earlier with L-012 in NOX2-deficient mice (66). In this context, nonspecific probes that react with several different reactive species can have the advantage of probably giving more general information on a biological process (*e.g*., inflammation) than do selective probes. Indeed, when combined with LC/MS/MS-based analytical analysis of the nonreacted probe as well as its different types of reaction products, nonselective probes may be seen as multi-purpose probes that provide quantitative information about different reactive species, as exemplified recently with hydroethidine (88).

### Chemiluminescent nanoparticles and ROS imaging

**FIG. 3.**
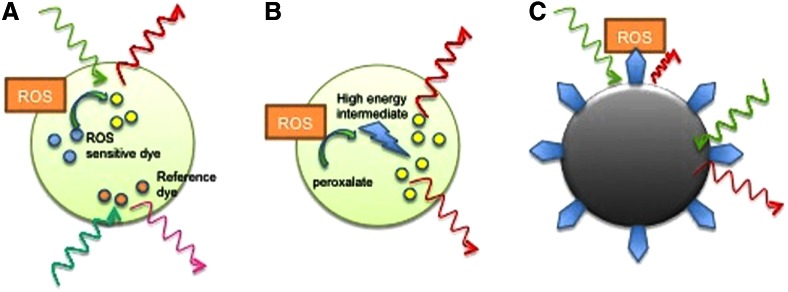
**Examples of NPs adapted for ROS sensing.** **(A)** Polymer-based NPs embedded with ROS sensing and reference fluorescent dyes; **(B)** Chemiluminescent NPs; **(C)** Metallic NP fluorescence quenching on oxidation of functionalized ROS-sensitive molecules (*blue*). Adapted from Uusitalo and Hempel (142). Reprinted with permission of MDPI. NP, nanoparticles. To see this illustration in color, the reader is referred to the web version of this article at www.liebertpub.com/ars

Nanoparticles (NPs) are particles with at least one dimension less than 100 nm. They have different shapes, unique physicochemical properties, and a high surface-area-to-volume ratio. NPs have several advantages over small-molecule probes used in cellular sensing and imaging (142). First, NPs have stronger luminescent emission due to the large number of molecular probes that can be loaded into each particle. Additionally, their high surface-area-to-volume ratio provides a higher probability for analyte detection. The NPs may also protect the sensory contents from external interference, such as undesirable enzymatic reactions and nonspecific uptake by proteins. Moreover, it is also possible to target NPs to cells and subcellular compartments by conjugating appropriate ligand moieties onto their surface, which will allow for enhanced targeting to cells and subcellular compartments. Encapsulation and conjugation of different molecules, such as luminescent probes, proteins, or DNA, provides infinite possibilities in NP design for specific functions. In view of all of the earlier mentioned properties, NPs are becoming widely used tools in the field of sensing and imaging (Fig. 3) (115, 119).

Recent advances in developing various luminescence probes have enabled monitoring of ROS in both cells and animals. NP-based luminescent ROS sensors and their applications are summarized in Table 1. Lee *et al.* (77) developed peroxalate-based NPs formulated from peroxalate esters and fluorescent dyes to image H_2_O_2_ *in vivo* with high specificity and sensitivity. Peroxalate NPs are capable of imaging H_2_O_2_ in the peritoneal cavity of mice during a lipopolysaccharide-induced inflammatory response. The same group has improved the method by reducing the size of the NPs and modifying their content to detect H_2_O_2_ at physiological concentrations (22, 78). Luminescent NPs have also been exploited for *in vivo* targeting and imaging of tumor tissues. In a recent study, chemiluminescent NPs were successfully developed to image H_2_O_2_ as a tumor signal molecule (18). Such probes improve the stability of peroxalates in aqueous systems and are sensitive to low, physiologically relevant concentrations of H_2_O_2_ within the physiological range. This way of monitoring H_2_O_2_ should be helpful for a clinical diagnosis of other ROS-related diseases.

## Ultrasound in ROS Imaging

Ultrasonic imaging has been applied in many studies to detect changes in functional blood flow and atherosclerotic plaque associated with oxidative stress (84). Although radical oxidants cannot currently be directly detected in a clinical setting with ultrasound, a variety of original methods are being developed to enable such detection. Proposed approaches vary widely, but all rely on the central principle behind clinical contrast ultrasonography—the high sensitivity of ultrasound to echoes from gas bodies.

Contrast-enhanced ultrasound detects strong acoustic echoes when the ultrasonic pulse encounters micrometric gas-bubble contrast agents. A very specific acoustic signature can be obtained from microbubbles when they are acoustically driven at levels resulting in a nonlinear response during the compression and expansion phases of the microbubble. Several, very different solutions for detection of radical oxidants have been proposed based on the ultrasonic detection of microbubbles that are targeted to specific ligands, which are generated by chemical reactions or produced by micromotors.

Feasibility to detect inhibition of NOX in advanced atherosclerosis has been shown in mice using targeted contrast microbubbles bearing ligands for endothelial cell adhesion molecules that are involved in monocyte recruitment (84). Lipid-shelled decafluorobutane microbubbles were targeted to P-selectin or VCAM-1 and detected with a clinical ultrasound system (7 MHz), 8 min after injection at regions of atherosclerotic plaque in the aortic arch of mice. Inhibition of NOX was associated with decreased targeted detection of P-selectin and VCAM-1. This targeted ligand approach is the basis for a large amount of research in ultrasonic molecular imaging, but it remains to be a relatively indirect approach to assess oxidative stress. A more direct, bio-sensing ultrasound contrast agent for ROS detection has been proposed based on chemical reactions that generate gas-forming molecules in the presence of radical oxidants (108). In the presence of radical oxidants, allylhydrazine oxidizes into 2-propenyl-diazene that spontaneously undergoes a retro-ene reaction to generate gas-forming nitrogen and propene molecules. Allylhydrazine encapsulated in phospholipid liposomes (APLs) were produced (60 to 110 nm in diameter) and injected intravenously in mice. Images of the liver obtained 10 min after APL injection with a 14 MHz Siemens Acuson Sequoia 512 clinical ultrasound system were shown to present 40% higher video intensity in mice with inflammation as compared with mice without inflammation. APLs were specific to the hydroxyl radical, and it was further demonstrated that ultrasonic detection of APLs is sensitive to radical oxidant concentrations as low as 10 μ*M*. Even more recently, micromotor converters (MMCs) have been designed to produce microbubbles when H_2_O_2_ is present (104). Tubular MMCs with a platinum-coated inner surface were constructed to break down H_2_O_2_ as fuel while expelling an oxygen-microbubble trail. When injected in an *in vivo* model for abscess in rats, contrast-specific imaging revealed increased image brightness.

## PET/SPECT *In Vivo* Imaging of Oxidative Stress Using Radiotracers

The nuclear medicine imaging techniques PET and SPECT are based on noninvasive detection of the distribution of radioactively labeled molecules (radiotracers), and they combine an exquisite sensitivity (down to the femtomolar range) with a relatively low spatial resolution (one to a few millimeters). After it has been injected intravenously, the radiotracer circulates in body fluids, interacts with molecules such as membrane receptors, transporters, enzymes, structural proteins, *etc*., and/or is transformed by local tissue conditions, for example, blood flow, pH, redox potential, *etc*. Over time, the distribution of the radiotracer is modified according to the molecular composition of different parts of the body, creating the contrast in PET and SPECT images. With an ideal, that is, diffusible/high-affinity/low nonspecificity radiotracer, the laws of molecular interactions that govern reversible binding or irreversible trapping apply and allow derivation of truly quantitative information from the images, such as the concentration of a target protein or the activity of a target enzyme. Unfortunately, any PET or SPECT radiotracer that binds directly to ROS species has not been described so far. However, radiotracers that can image events correlating more or less with oxidative stress are available, that is, in increasing relevance order: (i) glucose consumption, (ii) cellular retention depending on the cytoplasmic redox potential, and (iii) radiotracers targeting ROS scavengers and the mitochondrial complex I-IV.

### Imaging glucose consumption as a surrogate of oxidative stress

The radiotracer that is most widely in use is [^18^F]Fluorodeoxyglucose (FDG), a glucose analog transported into the cells principally by GLUT-1 and GLUT-3. FDG is trapped in the cell cytoplasm after its phosphorylation by hexokinase to FDG-6-phosphate. The rate of radioactivity accumulation reflects local glucose consumption, and PET imaging with FDG is used universally for imaging glucose-avid tissues such as the brain or tumors. Jung *et al.* reported an indirect link between FDG uptake and ROS concentrations in cancer cell lines and tumor-bearing mice (61). They observed a parallel reduction of 30–50% of FDG uptake and ROS concentration after administration of resveratrol at doses of 50–150 μ*M in vitro* and 100 mg kg^−1^ *in vivo*. The ROS scavenger N-acetylcysteine had the same effect, whereas ROS inducers had an opposite effect (20–40% increase) on FDG uptake *in vitro.* Resveratrol treatment decreased the expression of the membrane glucose transporter GLUT-1.

The report by Jung *et al.* suggesting a relationship between FDG uptake and oxidative stress remains to be confirmed by other studies. In fact, a number of separate studies tend to indicate that increased oxidative stress is associated with glucose hypometabolism in neurodegenerative disorders, (99). Thus, it is likely that FDG uptake and ROS production are indirectly linked to other co-occurring factors. Further studies are necessary to determine whether the possibility to image changes in ROS production using PET imaging is relevant to specific diseases and/or to particular pharmacological challenges.

### Radiotracers with redox potential-dependent cellular retention

Popular SPECT radiotracers for imaging tissue perfusion [76], such as [^99m^Tc]-HMPAO, [^99m^Tc]-HL-91, and [^99m^Tc]-MIBI, are redox couples that, depending on the redox potential of the medium, can switch from a reduced, lipophilic, membrane-permeable form to an oxidized, hydrophilic, nonmembrane-permeable form. These radiotracers have high octanol-water coefficients and cross cell membranes freely in a few seconds. Once in the intracellular space, they are oxidized in the cytosol by glutathione or reduced proteins and the radioactive signal builds up through trapping of the membrane-impermeable oxidized form, leading to radioactivity concentrations that are proportional to perfusion in the normally perfused brain or myocardium for [^99m^Tc]-HMPAO and [^99m^Tc]-MIBI, respectively [43–44]. Conversely, defects in tissue perfusion after stroke or myocardial ischemia appear as a negative contrast on scintigraphic or SPECT images. Interestingly, the trapping of these radiotracers is also impaired after oxidative stress, suggesting that [^99m^Tc]-HMPAO and [^99m^Tc]-MIBI can negatively image changes in the cellular redox state, although it is not clear whether the cause is a drop in glutathione concentration or a modification of the redox status (101). Sasaki *et al.* examined the redox potential in the brains of young and old male DBF_1_ mice using [^99m^Tc]-HMPAO, glucose transport, and metabolism using [1-^14^C]2-deoxy-D-glucose (2-DG), and mitochondrial electron transport function using [^15^O]O_2_ (121). They found a decrease of [^99m^Tc]-HMPAO brain uptake at 24 and 30 months of age, a late decrease of [^15^O]O_2_ uptake at 30 months, and a trend toward increased 2-DG uptake with aging. Blankenberg and colleagues (13) used [^99m^Tc]-HMPAO to evaluate the efficacy of a novel redox-modulating agent in patients with rare and fatal mitochondrial brain diseases, including Leigh syndrome, polymerase γ deficiency, MELAS, Friedreich ataxia, Kearns–Sayre syndrome, Pearson syndrome, and mtDNA depletion syndrome. Although no control group could be included for obvious ethical reasons and the number of patients was limited, they observed a significant correlation between clinical improvement after treatment and reduced ^99m^Tc-HMPAO brain uptake, suggesting that [^99m^Tc]-HMPAO may be a useful marker of redox state in brain regions under conditions of chronic oxidative stress.

### Radiotracers with hypoxia-dependent cellular retention

Radiotracer imaging of hypoxia is based on the principle of free diffusion according to plasma flow followed by specific trapping of the radiotracer in hypoxic tissues. Several radiotracers are based on nitroimidazole derivatives such as the fluorine-18-labeled fluoromisonidazole ([^18^F]FMISO). Once inside the cell, the nitro group of [^18^F]FMISO is reduced to a nitro radical anion that is immediately reoxidized by oxygen in normoxic conditions. Conversely, under low oxygen pressure, [^18^F]FMISO is not re-oxidized but undergoes further reduction by electron transfer, leading to reactive species that form adducts with proteins and nucleic acids. Since radioactivity is trapped in hypoxic conditions, [^18^F]FMISO administration produces positive images of tissue hypoxia, that is, the lower the oxygen pressure, the higher the radioactivity concentration. However, the relationship between uptake and hypoxia is not straightforward in all tissues because of the complex metabolism of [^18^F]FMISO and because of its slow clearance from normoxic tissue (102), whereas ^18^F has a half-life of less than 2 h. In attempts to obtain more suitable radiotracers, other nitroimidazole derivatives such as [^18^F]-, [^124^I]-, and [^123^I]-azomycin derivatives (IAZA, IAZGP, FAZA, respectively) (103) have been developed for PET and SPECT imaging, as well as non nitroimidazole compounds, including [^62^Cu]- and [^64^Cu]-PTSM, [^99m^Tc]-ATSM, [^99m^Tc]-HL-91, *etc*, have been developed. (5). Several of these compounds are commercially available and are in clinical use for the staging of tumors according to their hypoxic status and/or to assess radiotherapy- or chemotherapy-induced hypoxia. Consensus on the utilization of hypoxia tracers and on the correlation between their capacity to image hypoxia and ROS production remains to be defined.

### Radiotracers targeting ROS scavengers or mitochondrial complex I-IV

An “old” radiotracer that recently regained interest is [^99m^ Tc]-DTPA–glutathione ([^99m^ Tc]-GSH), a labeled derivative of the intracellular tripeptide glutathione that is present in all tissues where its physiological function is to neutralize ROS (31). The transporter of GSH is overexpressed in cancer cells, leading to higher concentrations of GSH in tumors, in particular during multidrug and radiation resistance and in metastatic cancers. It was recently reported that the uptake of [^99m^ Tc]-GSH is high in CT-26 colon cancer xenografted in mice with tumor-to-muscle ratios reaching 4.3 at 4 h, compared with 2.0 in inflammatory tissue with lower ROS levels (67).

**FIG. 4.**
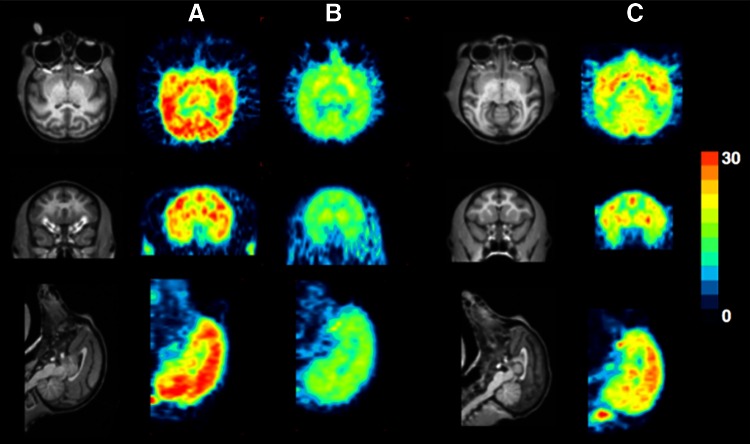
**Age associated neuronal impairment of MC I activity in the brain of living monkeys.** Typical MR and PET images of ^18^F-BCPP-EF in **(A)** normal young, **(B)** rotenone-treated young, and **(C)** normal old monkeys (*Macaca mulatta*). After infusion of vehicle **(A, C)** or rotenone at 0.1 mg/kg/h (**B**) for 1 h, PET scans were acquired for 91 min after ^18^F·BCPP-EF injection with sequential arterial blood sampling. The binding of ^18^F-BCPP-BF to MC-l was calculated using Logan graphical analysis with rnetabolite-corrected plasma input. Adapted from Tsukada *et al*. (139). Reprinted with permission of Springer. MRI, magnetic resonance imaging; PET, positron emission tomography. To see this illustration in color, the reader is referred to the web version of this article at www.liebertpub.com/ars

There have been continuous efforts by Japanese groups to develop radiotracers directly targeting the mitochondrial complex I-IV (MC I-IV) of the respiratory electron transport chain. Sasaki *et al.* have reported the labeling of [^11^C]idebenone, a coenzyme Q (CoQ)-related compound, and compared its biodistribution with that of [^11^C]CoQ_0_ (120). Although [^11^C]CoQ_0_ was better retained in cerebral tissue than [^11^C]idebenone, its clearance from the blood circulation was too slow for *in vivo* imaging of the brain given the half-life of carbon-11 (20.4 min). The authors concluded that further modifications of the isoprenoid side chain in [^11^C]CoQ would be necessary to obtain more suitable radiopharmaceuticals. Recently, Tsukada *et al.* developed fluorine-18 derivatives of BMS-747158–01, an inhibitor of the PSST subunit of MC I, among which [^18^F]F-BCPP-EF showed interesting pharmacokinetics in rats and monkeys, with rapid uptake into the brain and heart followed by gradual elimination (138). Specificity of the uptake was demonstrated using predosing with rotenone as a specific MC-I inhibitor. [^18^F]F-BCPP-EF was used to image the extent of neuronal damage in a rat model of brain ischemia, and the age-associated neuronal impairment of MC I activity in the brain of living monkeys (Fig. 4).

## Magnetic Resonance Modalities

### Basic principles and technical considerations

**FIG. 5.**
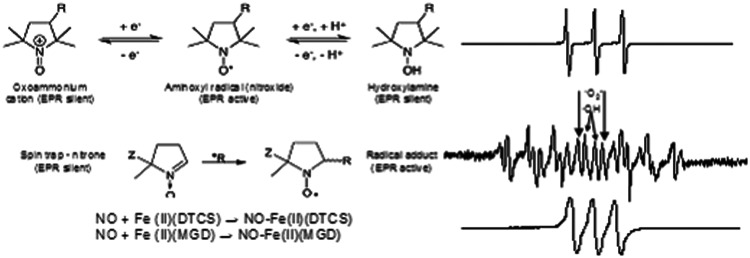
**Redox reactions associated with EPR-visible species (spectra on the** ***right*).** *Top row.* Nitroxides are stable in solutions, but not in biological systems, and can be sensors of redox status due to illustrated reactions. The basic structure can be the pyrrolidine or piperidine ring, which determines relative resistance to reduction (5-membered rings are generally more resistant). These two pairs: hydroxylamine/nitroxide and nitroxide/oxoamonium cation actually mimic cycling anti-oxidant and superoxide dismutase pairs. The group on the position 3 determines the behavior of the probe (solubility, lipophilicity, membrane penetration, *in vivo* clearance rate, *etc*.) and can be tailored to the needs. *Middle row.* Spin trapping. ROS are trapped with the nitrone trap converting them into a more stable form. Spectrum shows the ability of a trap DEPMPO (5-dietoxyphosphoryl-5-methyl-1-N-oxyde) to capture both superoxide and hydroxyl radicals that can be distinguished by characteristic spectral lines. *Bottom row*. Trapping of NO using DETC (diethyldithiocarbamate) or MGD (*N*-Methyl-D-glucamine dithiocarbamate) with different lipid solublility and membrane permeability. Adapted from Berliner and Fujii (7). Reprinted with permission of AAAS. EPR, electron paramagnetic resonance.

Electron paramagnetic resonance, EPR (or equivalently, electron spin resonance) is a spectroscopic technique that can directly detect paramagnetic species (species having an electronic spin due to the unpaired electron). However, there is very little to be observed by EPR in biological systems apart from some stable carbon-centered radicals, melanin, or transition metals. ROS such as superoxide or the hydroxyl radical are much too short lived to be detected by conventional EPR. Therefore, EPR detection of ROS can be accomplished by techniques that are not always direct. The scheme in Figure 5 explains the basic principles and strategies in ROS imaging. Injection of an EPR-visible nitroxide allows its detection in various organs *in vivo*. Endogenous ROS react with nitroxide, reducing it to an EPR-silent hydroxylamine and thus diminishing the EPR signal. The rate of reduction is the measure of the redox status of the tissue. But one has to be careful when interpreting such data, since the signal decay rate depends on several kinetic factors such as the distribution of the spin probe from the blood to the tissue and vice versa, urinary excretion through kidneys, and fecal excretion through liver and bile. Nevertheless, EPR monitoring of the decay rate of the injected nitroxide is the most efficient way to assess the redox metabolism *in vivo*, since one can use various nitroxides to unravel different processes. The alternative to this approach is to use acyl-protected hydroxylamine, which, introduced in the tissue, can be easily deprotected inside cells by intracellular esterases and then converted to the EPR-visible species by ROS-induced oxidation [58]. An entirely different approach to ROS imaging is EPR spin trapping, which is the “true” ROS imaging. The method relies on introducing a compound that will trap short-lived radicals and convert them into more stable paramagnetic compounds (Fig. 5).

Unfortunately, trapped radicals usually have rather complex EPR spectra that are not suitable for imaging. However, since multiple EPR lines do not affect overall paramagnetic properties of the compound, MRI has been successfully used in immuno-spin trapping (136).

Most of the basic principles of *in vivo* EPRI/EPRS were established in the 1980s (4, 7, 9, 26, 29, 60, 100, 111, 133). Much of this work has been stimulated by the discovery that nitroxides can report on the redox metabolism in cells and tissues and that the rate of reduction is highly dependent on the concentration of oxygen [see *e.g*., (131)]. Since then, several research groups have been developing specific spin probes with an adequate *in vivo* lifetime and other desirable properties, as well as instruments that are suitable for *in vivo* EPR. A standard commercial EPR spectrometer operating at 9.5 GHz (X-band) can at best accommodate a mouse tail due to nonresonant absorption of the electromagnetic radiation by the dielectric liquids in biological systems. Imaging of small animals, thus, has been performed at L-band (1.2 GHz) or even lower frequencies (around 700 or 300 MHz) (10). Commercial EPRI machines suitable for *in vivo* applications were not available until recently; hence, most researchers used and still are using home-made apparatus or a modification of commercial ones.

The realization that one can introduce metabolically responsive and relatively stable paramagnetic free radicals in the body and detect these processes promptly stimulated the introduction of MR in the area. MRI detects paramagnetic species indirectly, since they increase the relaxation rate of water molecules that can be seen by the enhanced signal on T1-weighted images. At the beginning, nitroxides were studied as potential clinical contrast agents, primarily for tumors, but recently they are more often used to study the redox state (14). MRI has no problems in imaging subjects of any size, including humans, since it operates in the frequencies of a few hundreds of MHz, but detection of ROS is indirect.

Both techniques have their advantages and drawbacks in *in vivo* ROS detection/imaging but the sensible simultaneous use of both is a way to employ the potential of these techniques, which has been demonstrated even for solutions (8). Namely, EPRI does not provide images of anatomy, it just shows the distribution of injected nitroxide within the body, and it does not have good spatial resolution. Conversely, MRI has excellent spatial resolution, but it gives little or no information on the paramagnetic species involved. Hence, using MR as an imaging modality and EPRS in combination can provide unique information (35, 37). It is also possible to use both techniques as imaging modalities and overlay EPRI, providing redox information apart from MRI providing anatomic information (15, 44, 57).

Numerous examples of combining these two techniques in oxymetry imaging can be found elsewhere (2, 81, 82, 83). There are also constructions of dual EPR/MR imaging machines (32, 39, 116). Probably the best way to fuse EPR and MRI into a single machine is to use the dynamic nuclear polarization (DNP or Overhauser effect) that uses a unique method for radical detection. An entirely different approach has been the combination of X-ray CT with EPRI in studying a mouse knee (11).

Perhaps the most powerful application of *in vivo* EPRI is measurement of oxygen (EPR oxymetry). This subject will not be covered *per se* due to limited space, although it is closely connected with the scope of this article. In addition, this subject has been extensively and regularly reviewed. What follows are characteristic examples that illustrate applications of magnetic resonance techniques in imaging ROS, particularly emphasizing how fruitful a combination of EPR and MRI can be in achieving optimal analysis of the investigated subject. A more comprehensive list of examples and a literature overview on EPR imaging of the oxidative stress can be found in the recent review (28), and more technical aspects of various EPR and MRI approaches with examples can be found in (56, 90).

### Examples of EPRI/MRI of ROS/RNS

Early EPRI images were rather crude (same as first MRI), and it took some 5–6 min to make crude 2D images using filtered back-projection with only eight projections resulting in low spatial resolution. (3, 111). It took full 45 min to obtain a complete 3D data set (60), which certainly limits temporal studies. However, this research stimulated further development, and today's machines are capable of producing 3D EPR images in around 1 min with up to 80 projections, where the actual performance depends on a selected task (34, 55, 155). Most research using EPRI and MRI was conducted using derivatives of TEMPO and PROXYL. In the beginning, carboxyl-PROXYL (3CxP, or then termed PCA) has been used (3, 4, 111), but later carbamoyl-PROXYL (3CP) became almost the universal choice for imaging, although different derivatives, such as hydroxylmethyl (HM-P) and others, have been used, especially in brain imaging (118, 151, 155). The proper selection of these probes with different properties such as *in vivo* half-life, membrane permeability, lipid solubility, *etc*. enables clarification of the location of *in vivo* ROS generation and redox status. As a rule, piperidine nitroxides have an *in vivo* half-life of a few minutes whereas the half-life of pyrrolidine is typically around 15 min or more. This is why pyrrolidines are generally used to image metabolism whereas piperidines are useful for probe circulation.

### Brain imaging (without tumors)

**FIG. 6.**
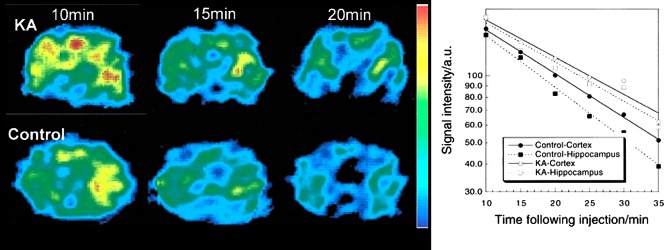
**EPRI of rat brain.** *Left*. The dynamic pattern of selected transversal EPR images of rat head 5 mm posterior to the bregma in the KA-treated and control groups at different times after injection of PCAM nitroxide. *Right.* Pharmacokinetic curves for brain regions. The cortical half-lives of PCAM in the control and KA groups were 18.0 ± 1.2 and 19.2 ± 0.7 min, whereas the hippocampal half-lives of PCAM in the control and KA groups were 10.4 ± 0.8 and 15.9 ± 0.7 min, respectively. Adapted from Yokoyama *et al*. (153). Reprinted with permission of Elsevier. KA, kainic-acid. To see this illustration in color, the reader is referred to the web version of this article at www.liebertpub.com/ars

The brain, due to its complex structure and function, has been a natural target for ROS EPR/MR imaging since the beginning of development of EPRI (60). This research has been accelerated by synthesis of the blood-brain-barrier permeable nitroxides (117, 140) and instrumental developments. Yokoyama *et al.* published a nice series of articles on various conditions induced in experimental animals (149, 150, 152–154). Figure 6 illustrates the basic concept of time-resolved brain EPRI (153). In rats with kainic-acid (KA)-induced seizures, the hippocampal half-life of nitroxide (PCAM) after KA-induced seizures was significantly prolonged, indicating impaired reducing ability; whereas the prolongation of the cortical half-life was not significant. These findings were confirmed by using an acyl-protected hydroxylamine that undergoes intracellular oxidation to nitroxides (150), showing that oxidative stress in the hippocampus and striatum in KA-treated animals is enhanced, but not in the cortex. Another set of studies, performed on the effect of various neuroleptics that are known to induce oxidative stress on the brain, revealed diminished ability of various brain areas in treated animals to reduce injected nitroxide (149, 152, 154). The study on intracerebral-reducing ability after acute stress in adult rats showed diminished reducing ability in rats that were subjected to neonatal isolation (154). Studies employing ischemia-reperfusion (I/R) injury induced by mid-carotid-artery occlusion using either only MRI (13) or EPRI/MRI combination (52) revealed slower reduction rates in brains that have undergone I/R. Another common way of altering the redox state is to induce septic shock, and it was shown that reduction rates of injected nitroxides are accelerated in brains of septic mice (36). Radiation is a certain way to induce vast changes in redox status, and various nitroxides have been successfully tested as potential radioprotectors (see (23) and references cited therein). In that study, nitroxides were used as both radioprotectors and indicators of redox status, and pharmacokinetics of nitroxides in brain, salivary gland, tongue, and oral muscle have been determined using MRI.

### Tumor imaging

**FIG. 7.**
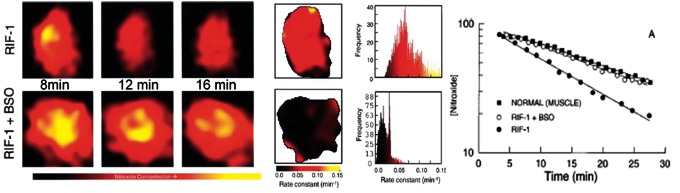
**EPRI of the thigh of mouse with implanted RIF-1 tumor.** *Left*: Selected EPR images of clearance of 3CP nitroxide in untreated and BSO-induced (agent for glutathione synthesis) tumors. *Middle*: Redox mapping of the tumor. Two dimensional mapping of pseudo-first-order rate constants and frequency plot of 3CP reduction rate constants. *Right:* The semilog plot showing the whole tissue clearance of nitroxide in tumors and normal muscle of contra lateral leg. Images of tumor and muscle used for the measurement of pharmacokinetic data were collected simultaneously on the same animals. Adapted from Kuppusamy *et al.*(74). Reprinted with permission of AACR. To see this illustration in color, the reader is referred to the web version of this article at www.liebertpub.com/ars

Due to their heterogeneous structure, tumors have been studied since the introduction of EPRI (9, 26). Redox status and oxygenation are important in designing therapy (especially radiotherapy) and/or in assessing tumor response to therapy. Tumors are heterogeneous in both aspects; hence, it is desirable to obtain spatially resolved images of nitroxide distribution and clearance simultaneously within the tumor volume as well as oxygenation, if possible. Various approaches employing the EPR/MRI combination, or individual technique and probe selection, have been used (37, 40, 54, 58, 73, 74, 98, 113, 132, 146, 156, 157). An example of tumor heterogeneity in reduction rates of nitroxide is given in Figure 7. It has been generally concluded that the reduction of nitroxides in tumors is faster than in normal tissue, irrespective of whether the reduction in tumors implanted in the muscle is compared with the muscle (54, 58, 73, 74, 146) or when gastric cancer is compared with normal mucosa (98). Faster bioreduction in tumors can be a consequence of an increased amount of endogenous reducing agents such as thiols [reduction was slower in both normal tissue and tumor in animals depleted with thiols (74, 146)], ascorbate, enzymes (Fig. 7). Chemically, nitroxides do not react with thiols, but altering the concentration of the thiol or changing the ratio of redox pairs has an impact on the clearance of the nitroxide; therefore, *in vivo* reduction of nitroxides also depends on the oxygen content and on the levels of GSH (74). Lack of oxygen, reflecting the well-known fact that reduction is faster in oxygen-depleted tissues, may also be responsible and tumors tend to have large hypoxic regions. Studies of tumors in animals breathing carbogen showed decreased reduction of nitroxides and decreased reduction heterogeneity with increased oxygenation (58), but a simultaneous study on reduction of nitroxides and direct oxymetry showed a rather poor correlation between these in normal air-breathing animals (132).

### Other organs

Skin is an ideal target organ for EPRI for several reasons. Imaging of ROS does not require a large penetration depth, so one can use the S-band (2.2–3.0 MHz) for *in vivo* specimens or even X-band for *in vitro* specimens, which results in improved sensitivity. Imaging does not require full 2D or 3D; once nitroxides are applied topically, a simple spectral-spatial 1D imaging with one gradient orthogonal to the skin surface is sufficient to obtain distribution of nitroxides and redox status in different skin layers. Surface loop coils are sufficient, that is, the whole object need not to be within the resonator, which allows EPRI of objects of any size, including humans. The potential of this technique has been nicely demonstrated in an *in vivo* study of human skin (46), which opens the possibilities of studying various skin pathologies, aging, or photo-damage. The effect of UV exposure on free radical production and redox status of the skin has been studied both *in vivo* and *in vitro* (45–47).

**FIG. 8.**
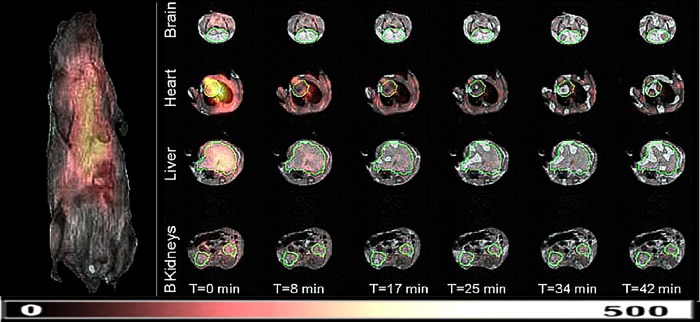
**Renderings of the superimposed 3D EPRI and 3D proton MRI of mice.** The color map is for the EPR intensity of the 3CP nitroxide probe distribution. *Left:* Coronal MR image of mice. *Right:* Transverse slices through different organs of the animal showing the temporal change of EPR intensity of 3CP. The *green* contour depicts the ROI used to calculate the average EPR intensity distribution of the probe later used to assess pharmacokinetics. Based on that, it has been found that mice exposed to second-hand smoking have diminished ability to reduce nitroxides in these organs. Adapted from Caia *et al.* (15). Reprinted with permission of Elsevier. To see this illustration in color, the reader is referred to the web version of this article at www.liebertpub.com/ars

Pharmacokinetics of nitroxides in abdominal organs (liver, kidneys, bladder) was first studied by *in vivo* EPRS (4) and EPRI (3, 111). The distribution and reduction/clearance of nitroxides demonstrated the feasibility of EPRI studies. But apart from having low spatiotemporal resolution, it has revealed difficulties in anatomical localization of different organs on EPR images. A decade later, it has been shown that this problem can be overcome by combining EPRI and MRI (44, 57) and that whole-body simultaneous measurements of pharmacokinetics and distribution of nitroxides can be performed on ten different locations within the body (57). These studies were performed to illustrate technical developments, and they were not aimed at investigating any particular pathology. An excellent application of a hybrid EPR/MRI machine has studied the redox status of different organs in mice exposed to cigarette smoke (Fig. 8). On the other hand, different important pathologies were studied using less technically demanding direct time-resolved EPRI. The study of mice liver showed a much slower reduction of 3CP in carbon-tetrachloride damaged liver than in the control (135). Another study of mice with hepatic I/R injury showed that CV159-Ca^2+^/calmodulin blockade inhibiting Ca^2+^ overloading has a profound effect on the liver-reducing ability (69). The I/R acute renal failure produced prolonged reduction of 3CP in kidneys (48), whereas it was much faster in the kidneys of diabetic mice (128). The latter study also showed that treatment with angiotensin returns the reduction to the control level, confirming the antioxidant properties of this drug. Another drug (Azelnidipine) has been studied in the murine hypertension model, and it has been found that it improves renal-reducing ability of free radicals, thus ameliorating the renal redox status (49). A somewhat different model of the investigated pathology was employed in a study of reducing activity of kidneys in Nrf2 transcriptional factor-deficient mice. The combination of deficiency and aging resulted in four times longer half-life of 3CP in the upper abdomen than in juvenile wild-type mice, indicating that low reducing ability may play a role in the onset of autoimmune nephritis (50). A set of hydroxylamine spin probes detecting site-specific production of the superoxide radical and allowing subcellular resolution and organelle specificity was developed and used *in vivo* on several organs. The detection mechanism is based on a rapid reaction of cyclic hydroxylamines with superoxide, producing stable nitroxides (25). These probes were applied *in vivo* on old rats, showed how ROS generation was significantly increased compared with their young counterparts in blood, skeletal muscle, lung, and heart, but did not change in intestine, brain, liver, and kidney (71).

### Imaging of trapped radicals

**FIG. 9.**
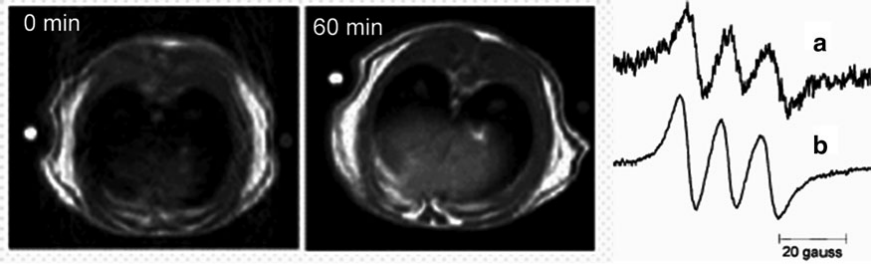
**LPS treated rats.** *Left*: T1W MRI images of the rat abdomen both before and after injection of the NO spin trap. *Right*: EPR spectra of trapped NO *in-vivo* on L-band **(a)** and on excised sample X-band **(b)**, demonstrating that trapped radical is NO and that MRI signal enhancement originates from NO. Adapted from Gallez *et al.* (37). Reprinted with permission of Wiley.

This attractive modality offers a possibility to image specific ROS as opposed to previous examples where the overall redox state was imaged. However, EPRI of trapped ROS is extremely difficult. First, the concentration of radicals is very low and it requires very high amounts of spin trapping agent to be injected (up to 100 mmol/kg), which raises the question of toxicity. Second, EPR spectra of trapped radicals usually contain numerous closely spaced lines of multiple adducts (Fig. 5) and it is almost impossible to isolate specific lines for imaging the selected adduct. Third, trapped products are not very stable, which narrows the time window for imaging. Nevertheless, imaging of trapped NO (Fig. 9) is a good example of how to combine *in vivo* EPR and MRI. The role of *in vivo* EPR is not to image radicals but to add the unique information on the nature of the radical species (fingerprinting), which actually enhances the tissue signal on T1W MRI, and a similar approach has been employed in brain imaging (36). Although there are no true images of trapped radicals, useful *in vivo* studies have been performed for employing specific EPR coils that detect signals only from targeted organs in assessing the usefulness of different traps in detecting radicals (134), NO generation in mice after cardiopulmonary arrest (75), and studies of simultaneous detection of oxygen and NO in the induced septic shock (33). Another example of successful *in vivo* detection of trapped radical is on the irradiated mouse, since irradiation produces a large amount of free radicals (43). Imaging of spin-trapped superoxide or hydroxyl radical could be improved by developing more resistant spin traps and using ^15^N-substituted probes, which will improve sensitivity and resolution by decreasing the number of EPR lines [see the nice review on *in vivo* trapping (64)].

### Dynamic nuclear polarization DNP-MRI (OMRI, PEDRI)

**FIG. 10.**
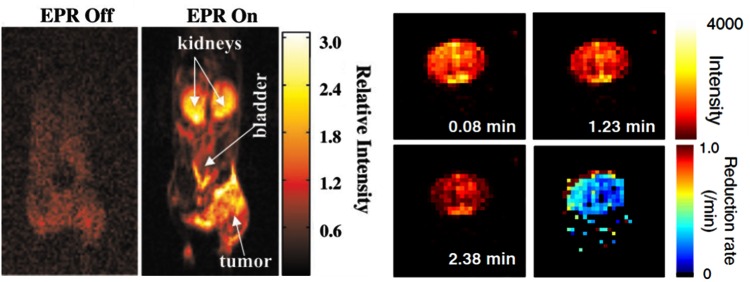
***Left*: Interleaved (“EPR off” and “EPR on”) OMRI images (coronal) of bearing SCC tumor on the** **right hind leg, demonstrating the OE and the diagnostic quality achievable at this low magnetic field of 15 mT.** The mouse was administered 3.8 mmol/kg triarymethyl radical by tail vein (72). *Right:* OMRI images of rat brain microinjected with neurodegenerative changes inducing agent (6-OHDA) into right hemisphere striatum. Redox status assessed 6 weeks later by the time-dependent OMRI signal of i.v.-injected methoxycarbonyl-PROXYL and the processed image showing the reduction rates in two hemispheres, demonstrating diminished reducing compatibilities in affected hemisphere. Adapted from Yamato *et al*. (147). Reprinted with permission of Elsevier. OE, overhauser enhancement. To see this illustration in color, the reader is referred to the web version of this article at www.liebertpub.com/ars

This technique deserves to be treated separately due to the unique detection mechanism and high potential for *in vivo* measurements, although the aim of these studies is the same as outlined earlier. DNP is not a simple overlaying of separate EPR and MR images obtained in a hybrid apparatus but a technique that includes parts of EPRI and MRI. Detection of radicals (unpaired electron spin) is based on a different principle. There are several DNP mechanisms (89), but most of the biological applications have been performed using the classical Overhauser effect, hence Overhauser MRI or OMRI. The first experiment using nitroxides and transfer to protons was performed almost 30 years ago (86) and was referred to as PEDRI (proton electron double resonance imaging). Briefly, the two-spin system (*e.g*., nitroxide/water protons) in the magnetic field is irradiated by RF at an EPR frequency of nitroxides (unpaired electron); magnetization is transferred to protons enhancing water proton NMR signal intensities; and the overall effect is detected by conventional proton MRI (86). This effect is completely different from classical enhancement of proton relaxation by nitroxides (theoretically 330 times higher). This is illustrated in Figure 10 on MRI of nitroxide-infused mice (72). Without EPR, RF irradiation nitroxides are invisible since classical enhancement is weak, whereas they can be clearly seen in the “EPR on” mode. Some nice examples (Fig. 10B) on the usefulness of this technique in imaging of the brain redox status have been published (147, 148). The OMRI combines the sensitivity of EPR with the advantages of MRI, thus presenting an ideal machine for ROS imaging. However, OMRI apparatus has to be home-built, which requires substantial skill and resources. The impetus for further development may come from the fact that *in vivo* DNP-MRI has an intrinsic capacity for molecular imaging of multiple species, similar to MR chemical shift imaging. By changing the frequency of EPR irradiation in DNP-MRI, distinct images of different radicals having different EPR spectra can be obtained, which has been demonstrated in an experiment where nitroxides labeled with ^14^N or ^15^N were simultaneously imaged (141). This approach was further extended, although in test tubes, to simultaneous imaging of free radical intermediates involved in the mitochondrial electron transport chain and radicals derived from vitamins E and K_1_ (53). Being able to simultaneously image species with a heterogeneous broad line having poor hyperfine splitting or species with complicated hyperfine splitting lines, which is impossible for standard EPRI, opens a host of possibilities, including metabolic imaging in various pathologies and imaging of spin-trapped radicals.

## Conclusions

The relevance of ROS in human physiopathology is now a well-established clinical notion (30). Reactive species are essential regulators in the physiopathology of disease; the knowledge of their concentration and local distribution with subcellular resolution is, therefore, a necessary clinical tool. This can be achieved by using different approaches based on the detection of redox couples, biomarkers that specifically bind to a redox species or that can modify their properties in the presence of ROS or some by-product of the oxidation (Fig. 1). The most investigated solution is the “photonic” one. A wide variety of fluorescent probes and fluorescent NPs in the visible range, specific for each redox couples, allow redox mapping with 200 nm resolution, according to the Abbe law. Recently, new optical techniques (Stimulated emission-depletion fluorescence microscopy, photoactivated localization microscopy, *etc*.) (80, 144) have been developed to break down the resolution limit up to 20 nm, but specific probes are needed and, at the moment, these are unavailable for redox detection. Instead, the development of redox-sensitive fluorescent probes allowed a quantitative detection of each component of each of the redox couples, representing a further step ahead toward the comprehension of ROS involvement in the human physiopathology.

For these reasons, fluorescence microscopy not only has become very popular in biomedical research activities (*i.e*., on cell lines) but also has found relevant translation applications in the histopathology of tissues from biopsies and in the investigation of dermal injuries (i.e., melanoma detection) (109, 110).

These techniques, however, suffer from two main drawbacks that hampered their potential translational applications: (i) the low penetration of the visible light into tissues (roughly 300 nm) due to the tissues' optical absorbance (mainly due to hemoglobin) and multiple scattering, and (ii) the toxicity of fluorescent probes. TPM with endogenous or chemo-selective probes offers an attractive approach to *in vivo* ROS detection, due to the probes' general compatibility with many biological systems without external activating enzymes and genetic manipulation. TPM for *in vivo* and internal tissue imaging by using endogenous probes is a very attractive option and has stimulated the development of TPM microendoscopes by using a gradient-index rod lens, miniature compound lens (68, 114). Otherwise, to avoid the low signal-to-noise ratio provided by endogenous or chemo-selective probes, and to increase the penetration depth, the use of TP-excited IR and chemiluminescent probes has been proposed.

Microscopy in the NIR-VIS region of the electromagnetic spectrum is therefore very promising, although it may suffer due to limited clinical applications, when large spatial areas have to be scanned. Indeed, if we are interested in ROS distribution on a large scale (*i.e*., on the whole organ of a human being), we need different approaches. These can be furnished by intriguing applications of techniques commonly adopted in clinical investigation.

Ultrasound-based techniques, as demonstrated for the APL bio-sensors, allow a detection of physiological concentrations of ROS, with a contrast and a spatial resolution that can exceed those provided by fluorescence and chemiluminescence-based contrast agents. Evaluation is possible on a rapid time-scale (minutes), and imaging systems are in widespread clinical use. However, although toxicity of APLs and MMCs remains a concern, functionalizing these agents allows a selective destruction of target tissues.

Toxicity is a minor feature for PET and SPECT that are noninvasive, but they do involve exposure to ionizing radiation. Besides its established role as a diagnostic technique, PET has an expanding role as a method to assess the response to therapy, in particular, cancer therapy, where the risk to the patient from lack of knowledge about disease progress is much greater than the risk from the test radiation. The principal concern in PET and SPECT redox imaging is the lack of radiolabeled molecules that bind to ROS, and that has limited success of nuclear medicine in the direct imaging of ROS. Nevertheless, indirect methods for imaging of glucose consumption, redox potential, hypoxia, as well as direct imaging of ROS scavengers and mitochondrial complexes have undisputable clinical interest. Considering the sustained efforts in development of new isotopes and labeling methods, PET and SPECT are poised to make significant contributions to the field in the future. Another limitation is the resolution of clinical and preclinical PET cameras (roughly 1 mm).

The EPR and MR imaging *in vivo* has become a powerful tool in experimental and preclinical studies of ROS/RNS or redox status on animals. Basic concepts are well understood, and directions for future developments are clear. On the instrumental side, further development of hybrid machines is an obvious goal. The problem remains that these machines are expensive and have to be more or less home-built. On the side of probes and traps, the development of those that show specificity toward certain ROS, specificity toward certain organs (*e.g*., tumors) and showing longer *in vivo* life time is required; some studies along these lines are already underway (1, 85, 107, 113). The major obstacle in the translation of these techniques to the clinic is the scarcity of centers possessing the equipment, and the lack of a focused concerted effort on certain clusters of widely relevant pathologies in which ROS may play a key role (*e.g*., Amyotrophic Lateral Sclerosis, Parkinson, Alzheimer, and other neurodegenarative diseases). Clinical application of EPR spectroscopy has been summarized recently (65), stating that the best perspectives are in oximetry and dosimetry ionizing irradiation. One can add certain potential in investigating skin pathologies, including melanoma, to the list, but, due to problems with penetration depth of microwaves, EPRI of human body analogous to MRI will never be possible. On the other hand, it has been successfully demonstrated that OMRI machines accommodating large subjects, including humans, can be built (72, 87), opening possibilities to combine all MRI capabilities with molecular specificity of EPR in diagnosis and treatment follow-up.

## Outlook

Potential clinical translation of ROS imaging is straightforward. Each of the presented techniques possess an attractive potential, but none of them can fulfill all the requirements in terms of sensitivity, spatial resolution, temporal resolution, probe availability, toxicity, and cost. The obvious solution is to perform parallel studies with two or more techniques or even better to integrate imaging modalities that may offer synergistic advantages over any single modality alone. Some hybrid imaging systems such as PET/CT, SPECT/CT, PET/MRI, and EPR/MRI have already been developed. Anatomical imaging techniques such as CT and MRI provide structural details; whereas functional modalities such as PET, SPECT, TP-fluorescence, EPR, and others provide insight into functional and metabolic aspects. Incorporation of anatomical and functional imaging in a common hybrid imaging platform should allow improved diagnosis, therapeutic planning, and follow-up studies.

## Abbreviations Used

APL: allylhydrazine encapsulated in phospholipid liposomes
EG: glutathione intracellular redox potential
EPR: electron paramagnetic resonance
FAD: flavin adenine dinucleotide
FDG: [^18^F]Fluorodeoxyglucose
FMN: flavin mononucleotide
Grx1: glutaredoxin 1
GSH: glutathione
GSSG: glutathionedisulfide
H_2_O_2_: hydrogen peroxide
HO_2_·: hydroperoxyl radical
HOCl: hypoclorous acid
HyPer: H_2_O_2_ fluorescent sensor
I/R: ischemia-reperfusion
KA: kainic-acid
MMC: micromotor converters
MPO: myeloperoxidase
MRI: magnetic resonance imaging
NAD: nicotinamide adenine dinucleotide
NADP: nicotinamide adenine dinucleotide phosphate
NADPH: nicotinamide adenine dinucleotide phosphate
NIR: near-infrared
NO: nitric oxide
NOS: nitric oxide synthase
NOX: NADPH oxidase
NP: nanoparticles
O_2_^•−^: superoxide radical
ONOO^−^: peroxynitrite radical
PEDRI: proton electron double resonance imaging
PET: positron emission tomography
PN1: peroxy naphthalene 1
PROXYL: 2,2,5,5-tetramethylpyrrolidine 1-oxyl
RNS: reactive nitrogen species
roGFP: GSH-sensitive green fluorescent protein
ROS: reactive oxygen species
rxYFP: GSH-sensitive yellow fluorescent protein
SHG: second harmonic generation
SPECT: single-photon emission computed tomography
TEMPO: 2,2,6,6-tetramethylpiperidine 1-oxyl
TP-FLIM: two-photon fluorescence lifetime imaging microscopy
TP-FRIM: two-photon fluorescence ratio imaging microscopy
TPM: two-photon microscopy
